# Commentary: Physical time within human time

**DOI:** 10.3389/fpsyg.2023.1096592

**Published:** 2023-06-15

**Authors:** Simon Prosser

**Affiliations:** Department of Philosophy, University of St Andrews, St Andrews, United Kingdom

**Keywords:** time, temporal experience, flow, passage, endurance, perception

## Introduction

It is exciting to see a growing interest in and new ideas concerning temporal experience from multiple disciplines. Some brief critical observations about each of the two target papers are presented in an attempt to further clarify the phenomenon behind one specific thread by Gruber et al.

## Comments on Buonomano and Rovelli

It seems odd to propose that neuroscience itself takes a view on the nature of time, let alone a view that is at odds with physics. Many sciences, especially those concerned with biological phenomena, can proceed as though classical physics were true, as though there were a single global present, and so on, for the reasons that Buonomano and Rovelli present, namely that, within their domain of inquiry, these classical claims hold approximately but to a high degree of accuracy. Even physicists sometimes proceed in this way, for example, when dealing with macroscopic phenomena or some of the practicalities of setting up experiments. It is another thing to suggest that any science other than physics is thereby in a position to take a view on the objective nature of time. Evidently, the only sense in which an opposing view of time essentially figures in neuroscience relates to the fact that neuroscience is in the business of explaining experience (among other things), and our experiences tend to suggest a world in which there is an objective present, time passes, and so on. However, great care is needed when inferring anything about the nature of time from the subjective character of experience. For experience could only inform us about the nature of time if there were a plausible mechanism that would make the characters of our experiences sensitive to the kinds of objective temporal facts in question. Yet, if no physical apparatus can detect the supposed passage of time, the global present, and so on, it follows that the brain, as a physical system, cannot do so either (see Prosser, 2016 for an extended discussion of this issue).

## Comments on Gruber, Block, and Montemayor

Gruber et al. covered a wide range of related topics very quickly, sometimes at the expense of clarity. It was hard to determine how to understand the “dualistic” proposed model. The talk of “two times” appears unnecessarily confusing, as it ultimately amounts to the familiar distinction between appearance and reality applied to time. Physics tells us certain things about time. Our experience of time suggests to us that time has a different nature than that suggested by physics. Physics is presumably right; therefore, either our experience involves some kind of illusion or there is something about our experience of time such that, even though the experience itself is veridical, it invites false beliefs about the nature of time. This is not happily described in terms of two different kinds of time, one inside the brain and one everywhere else. If someone's visual experience of a banana made it appear straight when it was curved, we should not say that there were two bananas, a curved one in the outside world and a straight one in the brain. There is no banana in the brain, not even a “mental” one; there are just numerous firing neurons and other physical processes that collectively constitute the experience of the banana and make it seem, to the subject whose brain it is, that the banana is straight.

Hartle's notion of an IGUS is doubtlessly useful in thinking about temporal experiences. It follows the principle that, if you want to know how something works, think about how to build one. One starts with a simple model and then gradually modifies it to bring it closer to the real thing. The metaphor of adding “gadgets” is not always helpful, however, since it suggests that the modifications in question involve simply adding further systems without fundamentally changing the underlying system. This is not automatically correct. In some cases, the gadget might alter the functioning of the underlying system. Moreover, in the case at hand, it sometimes appears that Gruber et al. interpreted the addition of gadgets as the basic IGUS having veridical experiences with a certain character and gadgets as adding a further, illusory character to the experience while leaving the underlying experience unchanged.

In some cases, it was not clear what was supposed to be illusory. Consider, for example, the discussion of experiencing motion and change. Gruber et al. seemed to follow Koch's (2004, p. 274) description of motion being “painted” onto an otherwise changeless “snapshot” (see Prosser, 2016, p. 125–127 for a discussion of what is wrong with this). In the case of phi or beta motion, where the stimulus consists of blinking static images, the experienced motion is illusory. However, in ordinary motion perception, where the stimulus is moving, no case was made by Gruber et al. for saying that anything is illusory. An object appears to be moving, and it is indeed moving in the straightforward sense of occupying different positions at different times.

A similar issue arises in the discussion of William James's observation that a succession of experiences is not sufficient for an experience of succession. The experience of succession is not usually construed as an illusory add-on to the succession of experiences. We typically experience succession veridically, insofar as “succession” consists of different things happening at different times. Both the “dynamic snapshot theory” (defended by Arstila, 2016, 2018; Prosser, 2016) and the views that it opposes are intended as theories of the generally veridical experience of motion and other changes and do not suggest that the contents of these experiences are in any way in conflict with the account of time given by physics (Prosser, 2012 suggests that there is an illusory “endurance” element in motion experience, but this is a separate claim and is not an essential commitment of the standard theories of change perception). Gruber et al. may have assumed that there is no motion or change in the “block” universe of modern physics and that experiences of motion or other change must therefore be illusory. However, the block theory does not rule out changes that consist of one state of affairs at one time and a different state of affairs at another time. Further research is needed to show that the experiences mentioned above concern anything beyond what has been described thus far.

## The role of endurance

Gruber et al. mentioned the philosophical notion of “endurance” and cited studies by some philosophers who proposed that the mind represents the experiencing subject, or other things, as enduring and that this has an important role to play in the illusory element of temporal experience. A brief suggestion will be presented here concerning the relevance of this to the illusory sense of “moving” through time and the extent to which this is compatible with the dualistic account.

An increasing number of philosophers (including those cited by Gruber et al., along with Prosser, 2012, 2016) have reasoned as follows. If we consider ourselves to persist by perduring, that is, by having different temporal parts at different times, this does not seem to allow for the notion of moving through time. Each temporal part remains at its temporal location and nothing changes. Then, perhaps, instead, our minds represent ourselves and perhaps other things, as enduring, such that the very same entity (and not merely parts of it) is located at one time and then another. This representation may be illusory, but it helps explain a sense of motion through time (this “sense” may or may not be strictly phenomenological. For example, it might arise from one's current sense of being at a certain location in time while remembering being at an earlier time). In terms of the dualistic model of Gruber et al., however, while the representation of oneself as enduring may be illusory, it is not clear what would count as the underlying veridical representation.

Let us consider this more carefully. Nothing can literally move through time. Moving through space means being in different places at different times. Thus, moving through time should mean being at different times at different times, but taken literally, this means nothing.

Moreover, the notion of being at one position in time and then another indicates that we must understand endurance in terms of being entirely located in one position in time (this is not the only way in which philosophers have construed ‘endurance,' but it seems essential here). However, an object that is located entirely at one position in the time series exists only momentarily; it does not exist at any other time. Such an object does not move through time. If presentism were true, and the world were unextended in time, then there would be a sense in which all objects would exist only at one time. Nevertheless, it is not clear why representing the world as though presentism were true should create a sense of moving through time since there would be no extended region of reality through which to move. Instead, there should be a constant change in properties. Therefore, on its own, the subjective endurance claim faces problems.

Consider, however, the possibility that even though there is only one real-time dimension, our minds have two separate ways of representing it. Let us call these time_1_ and time_2_. Then, it could at least appear to make sense, from the subject's point of view, to say that an object was first at one location in time_1_ and then at another location in time_1_, where “then” implies “at a later location in time_2_.” Thus, an important question for empirical study is whether the brain has two separate ways to represent time (see Hoerl and McCormack, 2019, for one possibility, though it does not seem a perfect fit).

Where would this leave the notion of endurance? A perduring object moves through space by having different temporal parts in different spatial locations but is entirely located in one spatial location at any given time. Given the model above, it might appear that an object could seem to move through time_1_ by seeming to have different temporal_2_ parts at different times_1_. However, this would still involve representing the object as located entirely at one position in time_1_ at any given moment in time_2_. If objects objectively perdure, what would be represented at each time would be a temporal part. However, either way, an object (or person) would be represented as though it existed entirely at one time, and hence endured, relative to time_1_.

At first glance, the distinction between time_1_ and time_2_ might appear to support the dualistic model. This would presumably depend on whether one represented time series could be construed as a veridical representation belonging to the simple IGUS, with the other added as an illusory “gadget.” However, it does not seem clear why the representations of time_1_ and time_2_ would stand in quite this relation.

## Author contributions

The author confirms being the sole contributor of this work and has approved it for publication.

## References

[B1] Arstila V. (2016). “The time of experience and the experience of time,” in Philosophy and psychology of time Studies in Brain and Mind, Vol. 9, eds B. Mölder, V. Arstila, and P. Øhrstrøm (Cham: Springer), 163–186. 10.1007/978-3-319-22195-3_9

[B2] Arstila V. (2018). Temporal experiences without the specious presen*t. Aust. J.Philo*s. 96, 287–302. 10.1080/00048402.2017.1337211

[B3] Hoerl C. McCormack T. (2019). Thinking in and about time: a dual systems perspective on temporal cognitio*n. Behav. Brain Sci*. 42, e244. 10.1017/S0140525X18002157 30251619

[B4] Koch C. (2004). The Quest for Consciousness. Englewood: Roberts.

[B5] Prosser S. (2012). Why does time seem to pass? Philos. Phenomenol. Res. 85, 92–116. 10.1111/j.1933-1592.2010.00445.x

[B6] Prosser S. (2016). Experiencing Time. Oxford: Oxford University Press. 10.1093/acprof:oso/9780198748946.001.000136389024

